# Exploring generational differences in the impact of stigma on mental health among people affected by leprosy in rural India: a qualitative study

**DOI:** 10.1038/s41598-026-55020-y

**Published:** 2026-05-29

**Authors:** Anil Fastenau, Noortje van den Bogaert, Srilekha Penna, Matthew Willis, Priyanka Chahal, Nimer Ortuño-Gutiérrez, Thomas Hambridge, Fabian Schlumberger, Patricia D. Deps, Sundeep Chaitanya Vedithi, Karli Montague-Cardoso

**Affiliations:** 1 German Leprosy and Tuberculosis Relief Association India (GLRA India), New Delhi, India; 2 Marie Adelaide Leprosy Center (MALC), Karachi, Pakistan; 3 German Leprosy and Tuberculosis Relief Association (DAHW), HQ, Wuerzburg, Germany; 4 Heidelberg Institute of Global Health (HIGH), University of Heidelberg, Heidelberg, Germany; 5 Department of Global Health, Institute of Public Health and Nursing Research, University of Bremen, Bremen, Germany; 6 School of Medicine, Dentistry & Biomedical Sciences, Queen’s University Belfast, Belfast, UK; 7 Department of Global Health and Social Medicine, Harvard Medical School, Boston, USA; 8 Damien Foundation, Brussels, Belgium; 9 Erasmus MC, University Medical Centre Rotterdam, Rotterdam, The Netherlands; 10 Department of Social Medicine, Postgraduate Program in Infectious Diseases, Federal University of Espírito Santo, Vitória, ES Brazil; 11 Department of Medicine, University of Cambridge, Cambridge, UK; 12 PLOS Mental Health, PLOS: Public Library of Science, Cambridge, UK

**Keywords:** Leprosy, Stigma, Discrimination, Mental health, Anxiety, Depression, India, Human behaviour, Public health, Quality of life

## Abstract

Leprosy continues to be highly stigmatised in India, resulting in the continuation of poor mental health outcomes for affected individuals and delays in seeking treatment. Whilst previous research suggests that the effectiveness of intervention strategies to reduce stigma and improve mental health can vary across generations, little is known about how leprosy-related stigma specifically impacts the mental health of different generational groups. This study, therefore, explored these generational differences in rural India. Through qualitative in-depth interviews conducted between June and July 2022 with 20 people affected by leprosy in Sitapur district, Uttar Pradesh, several themes related to stigma and its impact on mental health were identified using thematic analysis. These themes were categorized under broader umbrellas: conceptions, social interactions and behaviours, clinical management, and mental health manifestations. These findings highlight the complex interplay of societal, informational, and psychological factors influencing stigma and mental health outcomes. Understanding how stigma impacts the mental health of individuals affected by leprosy is a complicated and multifaceted challenge. While the findings of this study suggest that knowledge and beliefs play a significant role in shaping how younger and older generations experience stigma in rural India, the implications for mental health are wide-ranging. This work also has broader implications for other remote settings and diseases within similar contexts, highlighting the need for further research to better understand these dynamics.

**Supplementary Information:**

The online version contains supplementary material available at 10.1038/s41598-026-55020-y.

## Introduction

With 107,851 new cases in 2022–2023 alone, India continues to account for the highest number of newly reported cases of leprosy globally^1^. Leprosy, also known as Hansen’s disease, is a chronic infectious disease affecting the skin and nerves, causing discoloured, faded patches of skin, nodules, or growths, and painless swelling or lumps on the face or earlobes^2^. Early detection and treatment is crucial to reduce long-term disabilities such as loss of sensation, muscle paralysis, deformity, and blindness^3^. Leprosy is not highly contagious, and most adults are not susceptible to infection due to an effective immune response^4^. Despite free access to effective Multi Drug Therapy (MDT) treatment, the prevalence of leprosy remains high in India^1^. The existing stigma that is associated with the disease is a large contributor to this high prevalence, as it creates a barrier for people affected by leprosy to seek help, which thereby delays detection and the initiation of treatment^3,5^.

Concepts of stigma are commonly described across several interrelated domains, including perceived, anticipated, enacted, and internalised stigma. These dimensions are widely recognised in stigma research and have been discussed extensively in foundational work by Link and Phelan^6^ and by Corrigan and colleagues^7^.

In the context of infectious diseases, these domains have been applied to better understand how stigma manifests and affects health outcomes. For example, Mukerji and Turan applied this classification to examine stigma experiences in tuberculosis^8^.

Building on this body of work, the present study draws on these established stigma dimensions as an analytical lens to explore how stigma impacts mental health across generational groups.The effectiveness of intervention strategies for reducing the impact of disease stigma on mental health in people affected by leprosy has been suggested to potentially vary across age groups^9,10^. However, to our knowledge, current interventions to reduce stigma lack strategies that are specifically tailored for younger generations (18–29) and older generations (30+)^11^. Investigating the effectiveness of a particular approach is difficult, because little is known about the specific effects of stigma within these generational groups. Therefore, the aim of this study is to investigate if and how stigma impacts the mental health of young adults and older generations affected by leprosy in rural India. The study will also assess whether there is a difference between the two age groups and identify potential protective and risk factors for mental health. This qualitative study aims to provide in-depth lived experience insights toward improving the mental health and wellbeing of people affected by leprosy by focusing on hard-to-reach villages in a leprosy endemic district of Uttar Pradesh (UP). By exploring the role of stigma in shaping the mental health of individuals affected by leprosy, this study aims to provide a much-needed nuanced understanding that will aid program implementers in optimizing resource use for effective interventions to reduce stigma and improve mental health. The findings also have the potential to be applied to other remote or under-resourced settings and to addressing stigma associated with other diseases in similar communities, broadening the scope of their potential impact.

## Methods

### Study design

This research was conducted as part of the first phase of the German Leprosy and TB Relief Association (GLRA) India’s larger intervention study, “Challenging Stigma through Engaging People Affected by Leprosy in Innovative Methods of Storytelling,” aimed at empowering individuals affected by leprosy in Sitapur, India. The primary objective of the larger implementation research project is to reduce leprosy-related stigma in Sitapur, India by encouraging participants to share their personal experiences through storytelling. This broader research employs a cohort design with a mixed-methods approach and includes two measurement points—pre- and post-intervention.

For the current study, however, only the pre-intervention period was examined, giving it a cross-sectional design and employing qualitative methods exclusively. Conducted in close collaboration with and under the supervision of GLRA India, the study involved one-on-one semi-structured in-depth interviews where participants recounted their lived experiences. Additionally, questionnaires were administered to capture further and specific insights into participants’ health and experiences with stigma. The interviews incorporated both open-ended and closed questions to facilitate in-depth responses.

### Study location

Sitapur, an Indian district located in the state of Uttar Pradesh, was chosen as the study area of this research. Sitapur is a predominantly rural district located in the state of Uttar Pradesh, India, with an estimated population of approximately 4.4 million people. Uttar Pradesh consistently reports one of the highest numbers of newly detected leprosy cases in the country, and Sitapur is classified as an endemic district under India’s National Leprosy Eradication Programme. The district was selected due to its sustained burden of new case detection and the presence of GLRA-supported leprosy control activities, providing access to individuals currently undergoing disability prevention and medical rehabilitation care. This district was chosen as the study site due to the high number of new leprosy cases within UP. The district consists of multiple villages that are located five to ten kilometres apart. Interviews were conducted in the community centre of the village named Tappa Khajuria due to its central position and convenient accessibility for participants coming from other villages. Moreover, precautions were taken to ensure privacy and confidentiality during the interviews.

### Study population

Participants included in this study were adults with a confirmed diagnosis of leprosy. Seven interviews were conducted with individuals affected by leprosy between the ages of 18 and 29 (2 women and 5 men), and thirteen interviews with people between the age of 30 and 70 (5 women and 8 men), leading to a total sample size of twenty participants. In India, ‘youth’ is considered as 15–29 years, as defined by the ‘National Youth Policy of India’^12^. This study included only individuals who had attained the age of majority, defined in India as 18 years and above. For the purpose of analysis, participants were grouped into two generational categories: those aged 18–29 years, referred to as the “younger generation,” and those aged 30 years and above, referred to as the “older generation.” This approach facilitates an exploration of generational differences, which may offer broader relevance and applicability to understanding stigma and mental health dynamics across age groups. Eligible participants were adults affected by leprosy, with or without disabilities, who were receiving disability prevention and medical rehabilitation (DPMR) services under GLRA India’s integrated project on leprosy, lymphatic filariasis, and WASH in Sitapur district between 2021 and 2022. Participants had to be willing and able to provide informed consent, be able to communicate with the interviewer directly or through an interpreter and be available to participate in the broader two-year GLRA India study. People with all levels of disability, grading 0, 1, or 2, as defined by the WHO^13^, were eligible.

### Sampling strategy

A systematic sampling method was used to recruit the participants of this study after attaining the official ethical clearance (see below). Sampling was carried out by a local GLRA-India team. Data on leprosy patients was acquired from the GLRA India’s project registry. Those who met the inclusion criteria of being managed for leprosy between 2021 and 2022 were compiled. Thereafter, the local team visited participants at their homes to introduce and explain the two-year study. Those who were willing to sign the consent form and met further inclusion criteria were enrolled in this study.

Given the exploratory qualitative design of this study and its focus on in-depth lived experiences, a relatively small sample was considered appropriate. The aim was not statistical representativeness but conceptual depth and thematic saturation within a defined rural setting. After twenty interviews, no substantially new themes emerged. The sample size is consistent with qualitative research aiming to explore meaning-making processes and stigma experiences in-depth rather than to quantify prevalence.

### Interview guide

The interview guide of this study was adapted from the Infolep’s validated qualitative research on stigma guide^14^.This guide contains multiple sections and open-ended questions. Based on the objectives of the two-year study by GLRA-India, questions were selected and modified according to the study context. The first version of the interview guide was prepared by the principal investigators and co-investigators of the larger study. Thereafter, the interview guide was discussed with the researchers of this study. Questions were added to cover the specific research objectives of this particular study. The interview guide was created over a course of nine weeks.

### Data collection

Interviews were conducted between the 28th of June and the 1st of July 2022. Before arrival in Sitapur, participants had been recruited by the local GLRA-India team after attaining the official ethical clearance for the study. All individuals who met the inclusion criteria were contacted again two days before the start of the data collection to confirm their scheduled timeslot for the interview. Both the consent and the demographic information were obtained before the interview for timesaving purposes. The interviews were conducted in Hindi. Close to half of the participants spoke a local dialect, for which a regional GLRA-India staff member had to be present to interpret between the interviewers and the participants. Emotional responses and other non-verbal communication were closely observed and documented alongside the responses both during and after the interview. All interviews were recorded and transferred to a digital file at the end of each day. Interviews lasted between 25 and 40 min.

### Data analysis

The audio files of the interviews were transcribed by several individuals who spoke both Hindi and the local dialect. The scripts, initially written in Hindi, were translated into English by local professional translators. The field notes taken during and after the interviews were used to identify themes. The method of thematic analysis was applied to study the scripts. To identify recurring topics, ideas, and conceptual patterns, or themes, a six-step process developed by Clarke and Braun^15^ was followed. Specifically, the analysis followed the six phases outlined by Braun and Clarke^15^. First, researchers familiarized themselves with the data by reading and re-reading the transcripts and field notes. Second, initial codes were generated systematically across the dataset using ATLAS.ti 22 to capture meaningful units of text. Third, codes were organized into potential themes by identifying patterns and relationships across interviews. Fourth, these preliminary themes were reviewed and refined to ensure coherence within themes and clear distinctions between themes. Fifth, themes were clearly defined and named to reflect their conceptual scope. Finally, the report was produced by selecting illustrative quotations and integrating the thematic findings with the study objectives and conceptual framework. A deductive analytical approach was selected, informed by widely recognised stigma dimensions (perceived, anticipated, enacted, and internalised stigma) as described in the literature^6,7^. These domains do not represent a single validated theoretical model but are commonly used as conceptual categories in empirical stigma research. The study aimed to explore generational differences within these established dimensions. Nevertheless, room was left for inductive insights to emerge during coding. Reporting of this qualitative study follows the Consolidated Criteria for Reporting Qualitative Research (COREQ) guidelines, and a completed COREQ checklist is provided as supplementary material.

### Researcher reflexivity

The research team included clinicians and global health researchers with experience in leprosy programmes in South Asia. Some members were affiliated with GLRA India, which may have influenced participants’ perceptions of the study. To minimise bias, interviews were conducted using a semi-structured guide, field notes were reviewed collaboratively, and themes were discussed across researchers from different disciplinary backgrounds. Reflexive discussions were held throughout the analysis to critically examine assumptions regarding stigma and generational differences.

### Ethical approval

This research received ethical approval from the Institutional Ethics Committee of Lepra Society India (07/LEPRA IEC/2022). Potential participants were identified and approached by the local research team through door-to-door visits. All individuals received detailed information about the study, and participation was entirely voluntary. Written and oral informed consent was obtained from all participants prior to the interviews. For individuals unable to provide a written signature, alternative methods such as thumbprints were used. Participants were informed of their right to withdraw at any time or decline to answer specific questions without any consequences. Given the sensitive nature of the topics discussed, a “Do-No-Harm” approach was followed^16^. Participants were informed about potential emotional discomfort and were given the option to pause or terminate the interview at any time. All data were anonymized prior to analysis and securely stored at the GLRA India head office. Recordings and transcripts were de-identified using unique participant codes, with the key stored separately in a password-protected file accessible only to the research team. Data will be stored until 2027 and then permanently deleted. All procedures adhered to the principles of the Declaration of Helsinki.

## Results

The interview responses were categorized into four main themes: Conceptions (preconceptions and beliefs, misconceptions, excessive precautions); Social Interactions and Behaviors (fear of loss of family, loss of marital prospects, they don’t need to know, fear of discrimination); Clinical Management (experiences with healthcare providers and disclosure & non-disclosure of diagnosis); and Mental Health Manifestations (depression and anxiety, and suicidal thoughts).

Throughout these themes, comparisons were made between designated age categories and gender. Older participants more frequently reported feelings of shame, guilt, and anxiety, as well as concern about discrimination and loss of family support. Conversely, younger participants more often employed strategies of autonomy and non-disclosure to navigate stigma. Gender differences also emerged, with female participants frequently articulating a deeper psychological burden, particularly related to fear of discrimination, loss of family, and their financial dependence on spouses, reflecting the influence of cultural and societal norms.

Further subthemes that emerged, such as self-isolation, avoidance, forced isolation, physical abuse and neglect, are described within the relevant thematic categories, providing a nuanced understanding of how leprosy-related stigma and its consequences differ across demographic groups within the community. Interpretations of these findings are presented in the Discussion section.

### Theme 1: conceptions

#### Preconceptions and beliefs

Respondents’ knowledge of leprosy prior to being diagnosed impacted their initial experience of anticipated and internalised stigma. A common phrase elicited by people in response to the question *What was your initial feeling when you got diagnosed?* was “*I was very nervous”.* However, the source of worry often differed based on their familiarity with the disease. The results indicate that more than one-third of the respondents had never heard of leprosy before they were diagnosed. Others were familiar with the disease but had little to no knowledge about its characteristics, mode of transmission and complications. Few people were aware of the disease and its social or physical implications.

Participants who had not heard of leprosy prior to diagnosis more frequently reported concerns about physical symptoms and livelihood rather than social stigma. Instead of being nervous or anxious about negative beliefs, people were worried about what would happen to their body, whether the repercussions of leprosy would prevent them from performing certain activities, leaving them unable to work, and these implications to their livelihoods, as the following quote demonstrates:

When I got the information about this disease from the doctor, I was only worried what would happen if my leg got damaged.

Woman aged 60

In contrast, respondents being familiar with leprosy had immediate fear of being discriminated. They often identified their diagnosis as *“Chu-achut”,* meaning “disease of untouchability”. Having experienced how inhabitants of their village had treated people affected by leprosy as “untouchables” in the past, newly diagnosed people were afraid of being discriminated against and isolated by society as well. Notably, women who had prior knowledge of leprosy were more affected by leprosy-related stigma and discrimination than their men counterparts. Women in this group frequently articulated how the stigma associated with leprosy impacted their mental well-being, often expressing feelings of shame, anxiety, and fear of social exclusion.

### Misconceptions

The common belief that leprosy is transmitted by touch is one of several misconceptions that respondents and people in their environment had. When asked *What do the people of the village think about this disease?* A respondent explains:

The people of the village think that distance should be kept from the person who has this disease, because according to them, this disease spreads by touching.

Woman aged 60

These false beliefs about leprosy run the risk of increasing the experience of stigma, as previous observation suggests that people influenced by their environment’s notion of the disease as ‘untouchable’ are more anxious about being stigmatised^17^. In addition, isolating an individual affected by leprosy by maintaining a distance is often a form of exacerbating existing stigma^8,18^. Moreover, given the low transmissibility of leprosy, it is also an *excessive precaution*^19^. Excessive precaution is a theme that is elaborated on later in this section. Participants frequently associated ongoing symptoms with continued infectiousness.

Our findings indicate that misconceptions about leprosy were more prevalent among older respondents compared to younger individuals. However, the data did not reveal significant differences in misconceptions based on gender. Other misconceptions identified relate to the reasons why people believe they got leprosy. By asking respondents how they think they can get the disease, it became apparent that many believe that everyone can get it; after all, they got infected as well. However, some people declared they did not know who could and could not get it, and several adult respondents believed that it was their fate to have gotten infected. One man was convinced he was getting punished by God for having committed the sin of marrying twice. Others declared:

No, not everyone can get it, but we don’t know. You get it if God gives it to you.

Woman aged 50.

And,

People say that this can be done by touching, the rest is a matter of their own fate.

Woman aged 65

Even though some were not able to explain what they had done to deserve “divine interference”, the belief that they were being punished for a sin committed in either this or a previous life remained frequent. This perception was particularly more frequent in older participants, irrespective of gender. Participants expressing beliefs of divine punishment also reported feelings of guilt and inferiority. Knowing one is not at fault may reduce a person’s internalised stigma, whilst, simultaneously, not knowing this may increase it^20^.

However, the results indicate that knowing leprosy is not a divine punishment, and instead a medical disease that everyone can get, does not necessarily reduce one’s internalised stigma. With his father being a doctor in the village, one respondent was able to nuance:

No, it cannot happen to everyone, but we feel that those who have less strength and capacity in their body, they can.

Man aged 22

Despite this young man seemingly knowing that people with a compromised immune system are more prone to getting infected, he remained very ashamed of his condition and sometimes felt as if he had let himself or his family down.

To continue, all respondents appeared to know that the disease is treatable. Some respondents had already finished their treatment and considered themselves cured, while others were still in the midst of their multidrug therapy (MDT) treatment, determined to complete it:

If there was no treatment, how can the medicine be working on me?

Man aged 58

However, doubts and worries were sometimes expressed about the effectiveness of the treatment due to misunderstandings regarding the correlation between certain symptoms and infection status. For instance, the majority of participants believed that as long as they still exhibited signs and symptoms of leprosy, they could somehow infect others. This reflected a general lack of clarity about the transmission and contagiousness of the disease after starting treatment. For example, some respondents thought that as long as certain nerves in their body remained numb, they would remain infected and therefore contagious. One individual even terminated his treatment prematurely because the lack of improvement made him think the medicines were not working.

This misconception was consistent across participants with no major gender or age differences observed. Notably, this belief was often reinforced by doctors’ recommendations to maintain distance for six months after beginning treatment. Furthermore, our study revealed that one persistent misconception was that an individual remains infectious even after completing MDT treatment. This misunderstanding induced significant stress, as illustrated by the following narrative.

Furthermore, the findings of this study demonstrate that misconceptions and/or healthcare providers’ instructions, or a lack thereof, have led to respondents and those around them taking excessive precautions. The story described in Box 1 illustrates challenges related to misunderstanding of treatment and prolonged separation. Furthermore, the healthcare provider’s instructions to maintain a physical distance from others were also very socially isolating for her. The distance made it difficult for her and her husband to connect, which negatively impacted their mental health.

**Box 1:** Nirmala’s story (aged 22).

Nirmala (not her real name) and her husband were unaware of the fact that after completion of her leprosy treatment, she will no longer be infectious. Neither of them had been told that they would be able to continue their life without having to live in separation. Living separately, meaning maintaining a physical distance as much as possible, sleeping in separate beds, not having sex, and the lack of prospect had been hard on their marriage. Nirmala expressed she had little interest and pleasure in doing things and was often feeling down, depressed and hopeless. Additionally, she said that she worries a lot because of the disease, and that this makes her feel sad. After our principal researchers informed Nirmala and her husband that they can live together as a family again, they expressed great joy and relief	

Although it is well established that leprosy is no longer contagious after the initiation of MDT and transmission risk is negligible after MDT initiation, a few of the respondents reported being advised by their healthcare provider to distance themselves for 6 months.

*Self-isolation*. Given the general guidance by doctors, self-isolation was a frequent theme of concern amongst respondents. On the one hand, precautions of isolation were taken to prevent the spread of infection, obeying clinical guidance. On the other hand, they served as a protection mechanism from being stigmatised by the community.

The study revealed that women, particularly younger women, were most affected by self-isolation or practised it more frequently. This behaviour often stemmed from a heightened fear of discrimination and societal judgment. The extent of self-isolation came forth when the same young woman was asked what people in the village think about leprosy, answering:

I do not know, because I do not come out of the house.

Woman aged 22

Isolating oneself from the outside world can be both a direct strain on one’s mental health and well-being, and an indirect burden. Moreover, self-isolation was an indication of anticipating stigma from the community.

Self-isolation was reported more frequently among younger female participants.

*Avoidance*. If people in the village were aware that a person had leprosy, the respondent often reported being avoided as villagers *“keep a distance”*. This was a recurring theme, especially among older participants, with older women highlighting this experience more frequently. A woman explains how her neighbours refused to see her:

Respondent: “Before coming to know about this disease, neighbours used to come up to my house, but when the neighbours came to know about this disease of mine, they started keeping a distance.”

Interviewer: “How did you feel seeing the behaviour of your neighbours towards you?”

Respondent: “I expected this behaviour from my neighbours, but I did not like their behaviour.”

Woman aged 60

Similarly, to this lady, respondents expressed feeling *“bad”* when they were avoided by people in the community. This stigmatising behaviour induces internalised stigma, and more specifically, makes someone affected by leprosy feel ashamed of their disease.

This is further demonstrated by another older woman:

I do not go out often, but when I go, they stay far away.

Woman aged 50

These findings highlight that avoidance was predominantly experienced and expressed by older participants, particularly older women, who often faced both social exclusion and heightened feelings of shame. This generational and gendered difference underscores the compounded stigma experienced by older women, further impacting their mental well-being.

*Limitations of access to community resources*. Another excessive precaution that originated from the misconception of how the disease spreads by touch led to a woman not being allowed near the water tap:

*The people of the village did not let me fill water, the tap is also the government’s, but we cannot fill water.*

Woman aged 45

This respondent was *forced* to stay away from the tap because the villagers believed she could contaminate the water. Forced isolation is a theme that reappears later in this section when discussing (fear of) loss of family.

### Theme 2: Clinical management

They often referred to their condition as “*skin disease”* and explained that this diagnosis and a medication prescription were all they had been given by the healthcare provider when they went to the hospital. Without any significant differences in age or gender, many participants reported that clinicians intentionally withheld their diagnosis, either by avoiding explicitly naming the disease or by using vague terminology such as "skin disease." One young respondent describes:

I went to show the doctor, and then the doctor told me to get treatment for 6 months, and he did not tell me what the disease is.

Man aged 19

Apart from sometimes feeling ashamed of his skin condition, this young man was not worried that other people would find out he has this disease, nor had people ever tried to avoid him. His responses indicated that he does not experience internalised stigma nor anticipate any type of stigma related to leprosy from his community. This is because his “skin condition” has never been labelled as a disease of untouchability, therefore remaining unaware of the stigmatised beliefs surrounding it.

There were also respondents whose doctor had not disclosed that they had leprosy, but who later became aware after being told by people from their village, as shared by this elderly man:

When I went to the doctor to get medicine, the doctor said that its treatment will last for six months, […], but the doctor did not tell me the name of this disease. The people of the village and the neighbours have called it Leprosy.

A man aged 67

Responses from interviews indicated that people were purposely withheld from their diagnosis. However, this cannot be known with certainty, as other possibilities include that the respondent was unable to comprehend the doctor’s diagnosis due to a lack of understanding, and the occurrence of an error in the transfer of information regarding the diagnosis. It is important to note that no major age or gender differences were observed in these instances. The decision not to disclose the diagnosis to the patient seemed to be primarily based on the doctor’s judgment and discretion. Moreover, during the interview, several young adults did not directly disclose that they had leprosy, such as this young man, saying:

I went to see the doctor. Then we came to know that this is a numb disease and treatment is going on. The doctor asked to get treatment for 6 months and nothing has been told about the disease.

Man aged 18

It was only much later in the interview, however, that this respondent used the word *“leprosy”* for the first time.

### Theme 3: Social interactions and behaviours

#### (Fear of) loss of family

Respondents often confided in family members about their diagnosis. While some disclosed to multiple family members, others chose to share their diagnosis only with their spouse. Generally, respondents disclosed their disease only to those living within the same household, with disclosure to family members living separately being less common. Responses indicated that many anticipated stigma from their family and were afraid of being discriminated against as a result. For instance, apart from her husband, this woman chose to disclose her diagnosis only to a select few who supported her:

Our relatives do not have any kind of information about this disease. Only my mother and brother are aware of this and their behaviour towards me is still the same as before they found out.

Woman aged 67

The research indicated that there were no significant generational differences in the overall experience of disclosure. However, the fear of losing family support was relatively more prevalent among older participants. Woman, regardless of age, were particularly affected by this fear. Women more frequently expressed concern about loss of family support. Responses from some participants indicated that family members were often supportive of their relatives when they told them they had leprosy. When being asked *How is the behaviour of the family members towards you?* common responses were *“They are nice, there is no discrimination”*, *“Everyone in the house behaves the same as before they came to know about it”*, and *“It is good”*. One man says:

The people in the house have not said anything negative about it, they just told me to get the treatment and get cured.

Man aged 70

This man indicates that his family was completely OK with him after hearing his diagnosis and told him to just get the treatment and get cured. In contrast, the fear of losing family support was particularly pronounced among women across both older and younger generations.

*Forced isolation, neglect, abandonment, physical abuse.* However, family members being supportive was not a ‘given’. Some respondents in the older age groups, reported not being allowed to stay at home. Instead, they were asked to leave the house or forced to live in a small shed outside the family home. In some cases, the respondent and their spouse were asked to live separately from the rest of the family. Amongst this age group, respondents with visible deformities, as observed during the interviews, were more prone to being stigmatised and discriminated like this. The following story highlights how a woman was physically abused, neglected and forced to isolate by her family because of her disease.

### Loss of marital prospects

One of the things that the results showed, as depicted in the story written above (Box 2), is that leprosy can negatively influence marriage opportunities. This was revealed to be a deciding factor to not disclose one’s diagnosis, as demonstrated by the following respondent:

Respondent: “Only my husband knows about my disease. Except him, no one knows about this disease.”

Interviewer: “Why didn’t you tell your children about this disease?”

Respondent: “Even our children do not know about my disease because knowing about this disease can hinder their marriage. I never even fed the food from my plate to them.”

Respondent: ‘’Yes, I am afraid, if someone comes to know about my disease, how will my boys be able to get married, and the fear of this thing always remains with me.’’

[…]

‘’I feel ashamed because if someone comes to know about my disease, then it will affect the whole family, and the sorrow of this thing remains in my mind all the time

Woman aged 67

**Box 2:** Siri’s story (aged 70).

Siri (not her real name) is a 70-year-old woman currently under treatment for leprosy. She lives in a small village in Sitapur. Her fingers and toes are deformed, and skin patches are revealed when she rolls up her sleeves. Siri’s life changed for the worse after she got leprosy. When asked how she is doing, Siri starts to cry. She tells how her daughter-in-law beats her. How when visitors come to the house, she is being hidden, tucked away so no one will know she is there. No one in the family stands up for her, not even her son, whose wife physically abuses Siri. The family goes to great lengths to avoid anyone finding out she has leprosy, in fear of ostracization of the entire family. Siri is worried about the marriage prospects of one of her daughters. Having yet to marry, the family is afraid she would not be able to if people knew about Siri’s disease. She hardly gets any food at home. In order to feed herself, she goes to the temple every day and waits for people to make food offers to the Gods. When no one is looking, she takes the food and eats it. f	

This participant said she often feels as if she is letting her family down. Also, this passage illustrates that she anticipates stigmatising behaviour once people know about her diagnosis, because she indicates that her children would not be able to marry if people knew there was a case of leprosy in the family. This has been detrimental to her mental health and wellbeing. Also, she does not share her food with her children, because she fears they will get infected with leprosy if they eat the same food.

### They do not need to know

All young adults indicated that they had never experienced anyone avoiding them because of their disease. The fact that they all had no severe disabilities and very few visible physical signs of leprosy enabled them to keep their diagnosis private. This ability to maintain confidentiality about their condition appears to be a key factor in mitigating stigma.

Many young adults expressed that they saw no reason to disclose their diagnosis:

My lesions are not interrupting anyone or the job I do.

I am still the same person I was before; they do not need to know.

I am just dealing with it myself.

Even though some participants admitted to being occasionally worried that others might find out about their condition, they consistently reported experiencing less stigma. This finding underscores the importance of physical invisibility in protecting individuals from anticipated stigma and highlights how self-management and privacy can help reduce the social and psychological burden of the disease.

### Fear of discrimination

Many respondents did not disclose their diagnosis to people other than close family members, because they were afraid to get discriminated against by people living in their village. These respondents declare:

The villagers have a dirty feeling about this leprosy, and they discriminate the untouchables. We have not told anyone

Man aged 22

Everybody [in the family] knows. Nobody behaves differently. However, neighbours in the village were not told about this because they object. I did not tell about leprosy to them because then they do not let me sit next to them.

Woman aged 27

Both persons affected by leprosy quoted above indicate that they are aware that people in the village think leprosy is a ‘disease of untouchability’, and that villagers maintain their distance from those who have it. Both respondents that had heard about leprosy before, and those who had not displayed this same non-disclosing behaviour.

Generational differences were evident in how respondents managed stigma and discrimination. Older participants were comparatively more concerned about discrimination, which could partly be attributed to the fact that some of them had visible deformities, making it harder to conceal their condition. In contrast, young adults often navigated stigma by exercising autonomy and withholding disclosure. Their ability to keep their diagnosis private allowed them to avoid stigma and its associated social and mental health consequences.

Furthermore, respondents can be afraid of discrimination, whilst not afraid of people finding out about their diagnosis. This could be explained by the fact that some had a very low grade of disability, making it easy for them to hide their disease from others. This is not possible for everyone, for which villagers came to know about their disease despite efforts to conceal. Some made efforts to protect themselves by withholding the truth, as told by this man:

[…] when the villagers asked me, I said I do not know [about the disease] since they discriminate in the village on touch.

Man aged 67

Without other people knowing, a lot of respondents have never experienced any enactments of stigma and discrimination. However, those who did disclose that they had leprosy to their community, or those who were unable to hide their diagnosis due to uncoverable physical deformities, were often stigmatised by the people around them.

Gender differences were also clear in the results. Women were more profoundly impacted by the fear of discrimination than their men counterparts. This fear often affected their mental well-being, leading to feelings of shame, anxiety, and social withdrawal. Women more frequently reported fear of discrimination, shame, and social withdrawal. The persistence of fear of discrimination after disclosure was reflected in acts of self-isolation. Participants reported self-isolation and non-disclosure in response to anticipated stigma.

### Theme 4: Manifestations of mental health challenges

#### Depression and anxiety

Leprosy-related stigma caused feelings of depression and anxiety in our study population, as exemplified by this young respondent saying:

Yes, the mind remains depressed because of the disease, and the anxiety remains.

Man aged 19

Respondents reported having trouble sleeping, feeling hopeless, depressed, sad, and being less interested in things ever since they discovered they have leprosy. One participant, who felt ashamed and had been avoided because of her disease, explains how leprosy has affected her mental health and psychological well-being:

The way I was happy before, I am not happy like that after having gotten sick.

[…]

*I used to worry all the time because of this disease.*

Woman aged 60

The study found that depression, anxiety, and other mental health issues were prevalent across both generations and genders. However, these issues were more frequently reported by the older generation, including both men and women, who often faced a greater cumulative burden of stigma and the long-term impacts of the disease.

Internalised stigma, anxiety about other people finding out, and being stigmatised by one’s family or the community were a large burden for people affected by leprosy, which left them with a lot of stress and worry in their everyday lives.

Another significant burden that made respondents anxious and feel depressed was the fact that their livelihoods were threatened. Many respondents were not able to work anymore, because leprosy made them quickly fatigued, and the damaged nerves caused physical disabilities that took away the strength in their limbs. Consequently, they were not able to contribute to providing for their family. This was especially worrisome for respondents who used to be the main earners of the house. This concern was primarily raised by men. When being asked if he ever felt bad about himself or felt as if he had let himself or his family down, this man explains:

Yes, it is because I am not able to work.

Man aged 58

Hence, not being able to provide for the family not only directly affected people’s mental health and psychological well-being, but also had an indirect negative impact on providers for the family internally stigmatising themselves. Hence, not being able to provide for the family not only directly affected people’s mental health and psychological well-being but also had an indirect negative impact through self-stigma experienced by those who previously provided for their families. Men more frequently reported distress related to loss of their role as income providers.

### Shame/Guilt

The people affected by leprosy were often ashamed of their condition and sometimes expressed feeling guilty towards their family members. This woman affirms:

I feel embarrassed due to leprosy, but what can I do, I try to cover it up.

Woman aged 39

Being ashamed resulted in individuals withholding a disclosure of their disease. This woman, too, made an extra effort to hide her skin patches with a saree, to avoid people finding out.

The results suggest that shame and guilt could be both a direct consequence of stigmatised beliefs or developed only after being stigmatised by others. These feelings were particularly predominant among women, who often internalised societal stigma more deeply, impacting their mental well-being. Furthermore, older individuals expressed relatively higher levels of shame and guilt compared to younger adults, possibly due to generational differences in perceptions of stigma and their longer-term experiences with the disease. In contrast, young men reported the least guilt or sense of shame, often expressing more confidence and autonomy in navigating their diagnosis.

### Suicidal thoughts

The results show that some older generation respondents had thoughts about self-harm or experienced suicidal ideation due to the view that harm or suicide was preferable to the effects of leprosy. This man acknowledges:

When someone asks too much about the disease or its treatment, I feel it is better if my life ends than living.

Man aged 50

The findings of this study illustrated that suicidal thoughts were more frequently reported among older participants, reflecting the compounding impact of stigma and long-term social exclusion on mental health. Additionally, women tended to articulate these feelings more often than men. Suicidal thoughts were reported more frequently among older participants and women.

## Discussion

This study explored if and how stigma impacts the mental health of different generations and genders with lived experience of leprosy in rural India. The following themes emerged from our findings: conceptions, social interactions and behaviors, clinical management, and mental health manifestations. The historic conception of leprosy as a ‘disease of untouchability’ appears to remain a primary driver of stigma associated with leprosy in India, which has an adverse effect on the mental health of persons affected by leprosy. Respondents revealed the manifestations of stigma that they had felt, feared and encountered from family and community members, and their mental health consequences. This section first proposes and explains an adapted framework. Thereafter, the findings of the current study are explored in more detail, where references to other diseases are made. In addition, recommendations are made throughout the analysis. Finally, limitations of the study are discussed.

### Conceptual framework informed by established stigma dimensions

**Fig. 1:**
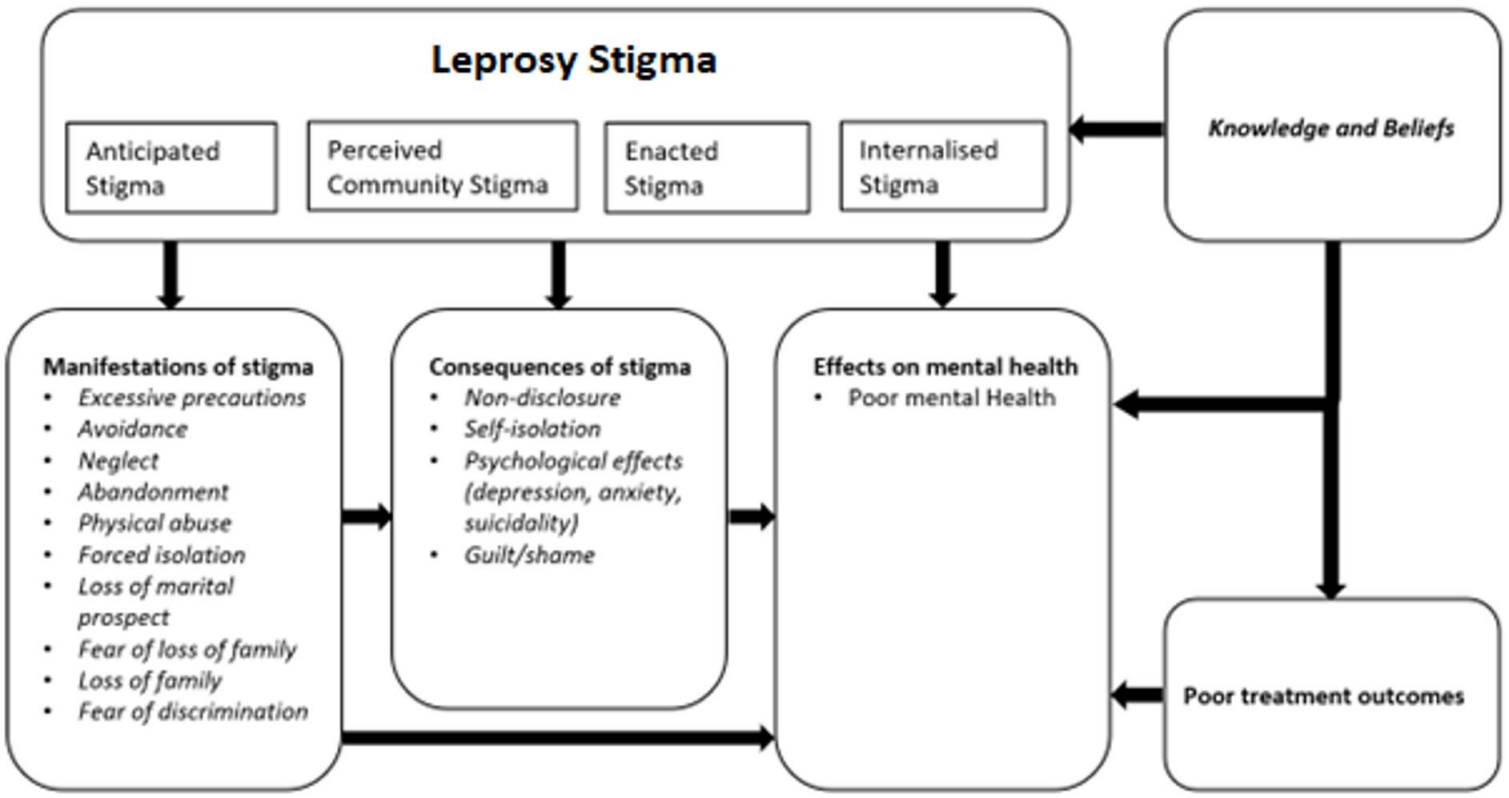
Proposed framework on the impact of stigma on persons affected by leprosy.

Based on the findings of this study, a conceptual framework is proposed (Fig. 1) to illustrate how different manifestations of stigma relate to mental health outcomes among people affected by leprosy.

This framework is informed by widely recognised stigma dimensions, perceived, anticipated, enacted, and internalised stigma, as described in the literature^6,7^. Rather than representing a modification of an existing formal theoretical model, it serves as a contextual and data-driven synthesis that integrates these established concepts with the empirical findings of this study.

The framework additionally incorporates contextual factors identified in the data, including generational position, visibility of disability, and healthcare communication practices, to better reflect the lived experiences of participants in rural India.

*Knowledge and beliefs* have been identified as themes in determining mental health outcomes of people affected by leprosy. The understanding that persons affected by leprosy, their family, and their community have about the disease is a significant contributor to a person’s experiences of stigma. Moreover, misunderstandings about the effects of the treatment can lead to poor treatment outcomes. Also, a lack of knowledge and false beliefs can cause unnecessary burdens, such as prolonged distancing, leading to marital issues, financial problems, and feelings of isolation. In contrast, *knowledge and beliefs* can also be protective factors for a person with lived experience of leprosy, either shielding one for or arming one against stigma or direct measures leading to poor mental health.

Several manifestations of stigma have been recognised within this study population. These manifestations of stigma may directly affect psychological wellbeing and may also trigger secondary consequences, such as social withdrawal or reduced livelihood opportunities, which in turn further affect mental health. What can be understood from the results is that many manifestations are interlinked and can overlap in certain aspects. Finally, this study identified consequences of stigma, which can be directly caused by stigma or by the manifestations of stigma.

### Leprosy-related stigma and strategies to reduce it

The results of this study suggest that leprosy remains a highly stigmatised disease in rural Indian society. In line with previous research^5,21,22^, experiencing stigma had a negative impact on the mental health of people affected by leprosy. A protective factor for experiencing the negative mental health manifestations associated with leprosy appears to be having a low grade of visible disability. Conversely, a high grade of disability could be a risk factor for stigma, which could be observed in Siri’s story. In a prior study, Adhikari et al.^23^, investigated the level of perceived stigma and the risk factors contributing to it in Nepal, and indeed found that participants with disfigurement or deformities had higher perceived stigma scores. Moreover, several studies have found that leprosy patients with higher disability were at greater risk of *depression*^22,24^. Tsutsumi et al.^24^ reported that the quality of life and overall mental well-being are likely to be worse for people with disfigurement compared to those without. However, in this study, it was difficult to tease out whether the adverse effects on mental health, such as depression and anxiety, were caused by stigma, because the results also indicated that people got mentally distressed when nerve damage prevented them from performing certain tasks. When this includes their work, one’s livelihood could be threatened due to the poverty the respondents lived in. This alternative explanation is supported by Adhikari et al.^23^ who found that participation limitations among leprosy patients had a negative influence on social life and mental health. Therefore, it is inevitable to advocate for certain stigma reduction interventions, including information, education, and communication (IEC), community-led initiatives, socioeconomic rehabilitation, integration of leprosy services within the health system, and cosmetic or surgical care, which can be effectively utilized in India to combat leprosy-related stigma^11^.

From a policy perspective, these findings underscore the need to strengthen stigma-reduction strategies within India’s National Leprosy Eradication Programme. This may include integrating structured psychosocial counselling into disability prevention and medical rehabilitation services, training healthcare workers in stigma-sensitive and person-centred communication, implementing community-based information, education and communication campaigns to address misconceptions about transmission and curability, and embedding routine mental health screening within leprosy services. Tailored strategies that account for generational and gender-related differences may further enhance the effectiveness of these interventions.

Under the theme of *non-disclosure,* it was observed that people decided not to disclose their diagnosis of leprosy to other people, so that they could not be stigmatised by others. For people with physical deformities, it was more challenging to conceal their diagnosis, making them more prone to outside stigma. While non-disclosure may function as a short-term coping strategy, it may simultaneously reinforce internalised stigma and fear of anticipated discrimination. This theme is also apparent in communities living with HIV/AIDS in which affected individuals were not willing to publicly discuss their status out of fear of being discriminated^25^. Other studies have observed that a common reason for non-heterosexual people not to come out for their sexual orientation is because they are afraid to be judged and discriminated by their community^26^. However, findings from previous studies dismiss this suggestion and indicate that *non-disclosure* is not an effective coping mechanism for persons affected by leprosy. Research by van Dorst et al.^22^ suggested that enacted stigma is not a determining factor in predicting poor mental health. These findings pointed out that the level of stigma is independently associated with the level of depression and mental health status amongst people with lived experience of leprosy. Moreover, a study by Tsutsumi et al.^27^ found that depression scores were higher in people with high levels of self-perceived stigma compared to people with low levels of self-perceived stigma, and that perceived stigma significantly affected all aspects of life, including mental health. A final protective factor observed in this study was being *withheld from the diagnosis* of leprosy by the clinician, which is elaborated on later in this section. However, researchers have demonstrated that several psychosocial interventions, including educational programs, counselling, cognitive behavioural therapy, and technology-supported approaches, can be effective in improving the quality of life and mental health of individuals affected by leprosy in India^28,29^. Thus, these psychosocial interventions should be an integral part of leprosy control activities in India.

To continue, *disclosure* and *misconceptions* elicited community stigma as well as internalised stigma. Respondents’ *fear of being discriminated* was attributed to these false beliefs. Hence, the mental health of people affected by leprosy could be improved by educating both patients and people in the community to decrease socially discriminatory acts. *Misconceptions*, including the notions of leprosy as incurable, extremely contagious, and transmitted by touch, have deep historical roots^30,31^. The history of other diseases suggests that focusing on removing these notions decreases stigma^32^. Initially, cancer, tuberculosis, and HIV/AIDS were highly stigmatised diseases, because a diagnosis used to be equated to death^32^. A hundred years ago, people who had tuberculosis were also much more stigmatised and shunned from society after it was discovered that the disease was not hereditary, but was instead transmitted through direct contact between people^8,33^. Therefore, combating misconceptions related to leprosy transmission and raising awareness about the curability of leprosy are essential steps for effectively controlling and ultimately eliminating the disease in India^34^.

The observed protective and risk factors might explain the careful observation that young adult respondents appeared to experience less stigma than older respondents. The results indicate that the young adults in this study were less likely to disclose their diagnosis to people in their community, had not been *avoided* or *forced to isolate* by others because of their disease, and all had a low grade of disability, in comparison to older adults. The way young adults have expressed that others “*do not need to know*” might suggest that their decision not to disclose comes less from a place of fear of discrimination, and more from a place of autonomy. However, even if young adults experience less stigma than older adults, this study found little evidence that would indicate they have different needs when it comes to intervention strategies, as hypothesised based on previous findings^9,10^. This study recommends that further research should be conducted in larger populations, urban settings and amongst people from different socio-economic backgrounds. In India, the age of marriage is significantly lower in rural communities than in urban settings^35^. Moreover, early marriage is associated with low socioeconomic status and education^35^. Most respondents of this study had a low income and had received little formal education. All in all, a lack of knowledge and misconceptions could be observed in both age groups, for which strategies should focus on increasing knowledge and removing false beliefs from society. In the Indian context, digital health tools can be used not only to diagnose leprosy but can also play a vital role in reducing the leprosy-related stigma and its impact on the mental health of persons affected by leprosy^36^.

### Role of the healthcare workers

Guidance for healthcare workers to improve their communication with persons affected by leprosy within a person-centred framework could play a role in decreasing stigma. Healthcare workers play a large role in transferring knowledge to leprosy patients. Especially in rural areas where access to medical knowledge is highly limited, people affected by leprosy are dependent on what is being told to them in the hospital. Healthcare workers should provide more information about leprosy to increase understanding. People currently remain limitedly informed, leaving them with a knowledge vacuum that is filled by one’s own or society’s misconceptions. To start, giving newly diagnosed patients better information, for example, about the fact that everyone can get infected, it does not spread by touch and is instead airborne disease, and it is not highly contagious, can help to guard people against stigmatised beliefs. Furthermore, monitoring and debriefing patients by having follow-up appointments give an opportunity to address potential misunderstandings. This also serves to lower the risk of poor medication adherence and can help reintegrate people into their life without leprosy.

In addition, the results suggested that correctly informing patients about the treatment procedure, including discussing the end of the infectious period as well as the importance of its completion, can minimise *isolation*. Healthcare workers instructed patients to distance for the duration of six months, leaving them isolated from their community longer than medically necessary. A potential reason assumed for this adjusted advice is to decrease the risk of treatment discontinuation. If a patient is told they are allowed to reunite with their family again after one week of medication, one could misunderstand that the disease is cured and that medicine is no longer necessary. However, Heijnders^5^ showed that isolation has severe consequences for a patient’s mental health and must be avoided to decrease the psychological burden of leprosy.

However, the public healthcare sector in India currently lacks the national capacity to offer effective and tailored communication and guidance that is needed to improve the knowledge held by people with leprosy. Therefore, this study recommends building or strengthening the capacity to meet this need. Moreover, resources should be allocated to providing context-specific and culturally sensitive psychosocial support and mental health care to both leprosy patients under treatment and people who have already completed MDT^29^. As has been observed in other chronic diseases, short-term psychotherapy and cognitive behavioural treatment may be beneficial. With limited resources available and dedicated to the field of leprosy, future research must investigate how current assets, as well as newly allocated resources, can be used cost-effectively and efficiently.

The results suggest that people are sometimes *withheld from their diagnosis* altogether. Instead, it is being referred to as “skin disease” or “skin patch”. In these instances, clinicians may have decided not to disclose to protect patients from stigma and its consequences. “Therapeutic non-disclosure” refers to a clinician’s decision to withhold diagnostic or prognostic information from a patient to protect him or her from perceived harm^37^.

According to Thilakavathi et al.^38^, which focused on the disclosure of leprosy in India, healthcare workers find it difficult to disclose the diagnosis of leprosy to patients and their family members. The health workers feared that stigma would lead to adverse effects for the patient or the family. However, the authors also found that those patients who were not disclosed to did not start treatment because they did not perceive their illness to be significant^38^. As suggested by Thilakavathi et al.^38^ stigma may spur patients to seek treatment and encourage adherence, because they would wish to be cured from a condition that is unwanted due to stigma. Therefore, even though the results of this study showed that being *withheld from the diagnosis* can be a protective factor for stigma, more work needs to be done to weigh the advantages and disadvantages of non-disclosure to patients of their diagnosis, considering the likelihood of others to tell them as well as the impact of removing an element of their autonomy. Although stigma may motivate some individuals to seek treatment due to fear of visible disability, this does not justify its presence, given its harmful psychological and social consequences. This study echoes the impact of leprosy-related stigma on the mental health and wellbeing of persons with lived experience of leprosy. Furthermore, this research demonstrates the strong presence of leprosy-related stigma in the Indian rural society, where the majority of global new cases of leprosy are reported. These findings emphasize India’s critical role in addressing stigma as part of global efforts to combat leprosy and its associated mental health challenges.

Additionally, the findings indicate that stigma-reduction strategies may benefit from greater attention to generational differences. Younger participants, who more frequently described non-disclosure and concerns related to employment and social identity, may benefit from confidential counselling and youth-focused awareness initiatives. In contrast, older participants, particularly those with visible disabilities, may require community-based reintegration support, family counselling, and structured psychosocial services integrated into rehabilitation programs. Although exploratory, these insights suggest the value of age-sensitive approaches within leprosy control efforts.

## Limitations

There were several limitations of this study. The sample size was small and within a relatively restricted region, and because of this, the analysis of the generational differences was challenging, though still informative and a good starting point for developing tailored support approaches. Further research should be carried out using a larger sample size across age ranges and in different communities. Additionally, certain words in the dialect were not understood by the researchers and transcribers, and very few parts of the recordings were not audible. However, given the small sample size and the potential impact on the robustness of the findings, all conclusions in this study were drawn solely from recordings that were clear and without any chance of misunderstanding. Finally, even though participants were well prepared by the local team before the start of the interview, and the researchers tried to build trust during the interview, this study was prone to sensitivity bias.

As a qualitative study conducted in one district of Uttar Pradesh, the findings are not statistically generalizable. Rather, they provide context-specific insights into stigma and mental health dynamics in rural endemic settings. The analytical generalization lies in the conceptual patterns identified, which may be transferable to similar socio-cultural and epidemiological contexts.

## Conclusion

This study underscores the profound impact of stigma on the mental health and well-being of individuals affected by leprosy in rural India. Our findings highlight that stigma, in its various manifestations, exacerbates psychological distress, leading to anxiety, depression, and, in severe cases, suicidal ideation. Misconceptions and lack of awareness about leprosy, including its transmission and curability, perpetuate these stigmatizing attitudes, emphasizing the urgent need for targeted educational and stigma reduction intervention strategies.

Moreover, potential differences in stigma experiences between generations reveal the influence of social and cultural dynamics, particularly concerning non-disclosure and coping mechanisms. While young adults often navigate stigma by exercising autonomy and withholding disclosure, older adults, especially those with visible disabilities, experience heightened stigma and its associated mental health consequences. Our study suggests that stigma reduction strategies must integrate culturally sensitive psychosocial interventions, community education, and healthcare worker training to mitigate the mental health burden of leprosy. Future research should expand to diverse socio-demographic settings and focus on tailoring interventions to address the unique needs of specific age groups and socio-economic contexts. By addressing both the social and psychological dimensions of stigma, stakeholders can advance leprosy control initiatives and improve the overall well-being of affected individuals. This research reinforces the importance of integrating mental health support into leprosy programs to achieve a more holistic approach to care and stigma reduction. At the same time, psychosocial interventions must be balanced in a way that affected individuals are aware of the seriousness of the condition and the risks of not seeking treatment. The goal is to have a well-informed population that also does not fear seeking support so that we observe a decline in new cases.

## Supplementary Information

Below is the link to the electronic supplementary material.

Supplementary Material 1

Supplementary Material 2

## Data Availability

Data is provided within the manuscript or supplementary information files.
