# Supplementary material for: Exploring generational differences in the impact of stigma on mental health among people affected by leprosy in rural India: a qualitative study
Source: Sci Rep. 2026 May 29;16:24611. doi: 10.1038/s41598-026-55020-y (PMC13454424; doi:10.1038/s41598-026-55020-y)
Supplement: Supplementary file 2 [file 41598_2026_55020_MOESM2_ESM.pdf]

Template Consent Form
(The consent form to be used in the study in English and all regional languages depending on the study participants)

Title of Study: **"Challenging stigma through engaging people affected by Leprosy in innovative methods of storytelling: an intervention study"**

Objectives:

General Objective

To assess the impact of innovative methods of storytelling through films on reducing internalised stigma and societal stigma related to leprosy and people affected by leprosy.

Specific Objectives

1. To examine the mental well-being, and level of internalized stigma amongst the people affected by leprosy through semi-structured in-depth qualitative interviews.
2. To investigate the improvement in the overall mental wellbeing, degree of empowerment and reduction in internalised stigma in the people affected by leprosy after participation in innovative storytelling interventions.
3. To determine the relationship between the mental wellbeing of people affected by leprosy and their participation in innovative storytelling interventions.
4. To examine the level of societal stigma amongst the community members towards people affected by leprosy through quantitative interviews.
5. To examine the impact of success stories of people with lived experience of leprosy through films in reducing the societal stigma.

Explanation of the condition: (explain the patient about the proposal in easy and understandable language)

A. Do I have to take part in the study?

   It is completely up to your wish to participate.

   If you think you want to help the researchers understand the challenges you face.

   The researcher will ask you a few questions related to the general awareness of the disease occasionally seen in your area.

B. What are the Times of sample collection?

   No sample will be collected.

   A researcher will ask certain questions about your awareness about the illness and will record your responses at a time convenient to you, preferably between 9AM to 5PM.

C. Who is doing this study?

GLRA India, an NGO that works for the welfare of disabled people in your area.

D. Number of subjects expected to participate:

Including you, we plan to record the views of 460 participants residing in different locations in the states of Uttar Pradesh and Madhya Pradesh.

E. What are the possible benefits in this study?

Your participation is crucial in the study, as the responses collected from you and other participants will benefit the health of the people in your community.

This will also help the National Health authorities in better planning of awareness campaigns in your area and health services at their centers.

F. What are the possible risks involved in this study?

As such, no risks are involved in this study. The researchers will not use your private information in any report. For the analysis of data, your information will be removed from the final data sheet.

However, if you are expressing your views over video then you might have concern that your community members may know your views. This will not happen, as your views expressed in the video will not be used in your state for awareness purposes.

G. Will this effect on the present services provided to me?

No, the services you receive either from GLRA or the government as such, will continue as before.

Even if you choose not to respond to any question, we will skip the question and move on to the next.

H. Is there any Compensation provided to me for study?

Usually the research projects do not have provision for payments to the study participants.

However, to compensate for your wage loss, we will provide a nominal amount in two installments.

In the beginning, an amount of Rs …. will be paid at the completion of the first interview and Rs ….. at the completion of 2nd interview in the …..month.

I. How will my samples be used?

No sample will be collected from you.
All your personal identifiable information will be removed from the data collected before analysis.

J. Will I be informed about the outcome/result of the study?

We will be glad to inform you of the result of the study if you are interested.
The results of the study will be available during the …. year of this data collection.

K. Will I be informed about the risk of discovery of biologically sensitive information?

No biologically sensitive information will be collected during the study.
Your identity will be changed, if you expressed your views in the video and the video will not be used for awareness purposes in your state.

L. What will it cost me to participate?

There will not be any financial implication on your participation. The researcher will come to you at a place and time convenient for you either at home or at the public health clinic.

M. Can I withdraw from the study at any point of time?

Yes, you have an option to withdraw from the study at any time OR you may even choose not to respond to any question that makes you uncomfortable.

The services you receive from GLRA or the government will continue as before.

However, in the interest of community benefits, we expect that you may like to continue your participation in the study.

N. Confidentiality:

The information collected from you is strictly confidential and is never disclosed to any other person or even the government.

The researchers will remove your personal information like name, etc from the final datasheet used for the analysis.

The data collected is strictly monitored by Principal Investigators and Co-Investigators to ensure confidentiality of the information received from you.

O. Consent to participate:

Your concurrence to participate in this research is welcomed.

I voluntarily agree to participate in this study.

Even if I agree to participate now, I understand that I may withdraw at any time or refuse to answer any question without facing any repercussions.

I was explained about the study, and I was given an opportunity to ask questions about it.

I understand that taking part in the study involves an interview.

P. Contact details:
   - For medical questions:
   - For questions about the study:
   - For questions concerning my rights as a participant contact:

Counselled by: ______________________________________

Designation: ____

Name of Patient / Parent	Signature / Thumb Impression
Place:	Reg. No.
Date:	
Address / Ph No:	

Name of the witness	Signature / Thumb Impression
Relation to the patient	
Place	
Date	
